# Variants in the Mannose-binding Lectin Gene *MBL2* do not Associate With Sepsis Susceptibility or Survival in a Large European Cohort

**DOI:** 10.1093/cid/civ378

**Published:** 2015-05-12

**Authors:** Tara C. Mills, Stephen Chapman, Paula Hutton, Anthony C. Gordon, Julian Bion, Jean-Daniel Chiche, Paul A. H. Holloway, Frank Stüber, Chris S. Garrard, Charles J. Hinds, Adrian V. S. Hill, Anna Rautanen

**Affiliations:** 1 Wellcome Trust Centre for Human Genetics, University of Oxford; 2 John Radcliffe Hospital, Oxford; 3 Section of Anaesthetics, Pain Medicine and Intensive Care, Imperial College London; 4 School of Clinical and Experimental Medicine, University of Birmingham, United Kingdom; 5 Hospital Cochin, Paris, France; 6 Department of Anaesthesiology and Pain Medicine, Bern University Hospital and University of Bern, Switzerland; 7 Barts and The London School of Medicine Queen Mary University of London, United Kingdom

**Keywords:** mannose-binding lectin, MBL, genetics, sepsis, association study

## Abstract

***Background.*** Sepsis is an increasingly common condition, which continues to be associated with unacceptably high mortality. A large number of association studies have investigated susceptibility to, or mortality from, sepsis for variants in the functionally important immune-related gene *MBL2*. These studies have largely been underpowered and contradictory.

***Methods.*** We genotyped and analyzed 4 important *MBL2* single nucleotide polymorphisms (SNPs; rs5030737, rs1800450, rs1800451, and rs7096206) in 1839 European community-acquired pneumonia (CAP) and peritonitis sepsis cases, and 477 controls from the United Kingdom. We analyzed the following predefined subgroups and outcomes: 28-day and 6 month mortality from sepsis due to CAP or peritonitis combined, 28-day mortality from CAP sepsis, peritonitis sepsis, pneumococcal sepsis or sepsis in younger patients, and susceptibility to CAP sepsis or pneumococcal sepsis in the United Kingdom.

***Results.*** There were no significant associations (all *P*-values were greater than .05 after correction for multiple testing) between *MBL2* genotypes and any of our predefined analyses.

***Conclusions.*** In this large, well-defined cohort of immune competent adult patients, no associations between *MBL2* genotype and sepsis susceptibility or outcome were identified.

## Abstract (precis)

We use a large cohort of immune competent adults to analyze the influence of *MBL2* genetic variants on sepsis susceptibility and survival. We find no significant associations with the 4 main functional single nucleotide polymorphisms in *MBL2*, or any combination of genotypes.

**(See the Editorial Commentary by Eisen on pages 704–6.)**

The incidence of sepsis is increasing and mortality rates remain high [1]. Sepsis was recently reported to be responsible for 1 in 20 deaths in England [2]. The frequency of hospitalizations with severe sepsis has increased relentlessly, more than doubling each year between 2000 and 2007, with sepsis complicated by organ failure accounting for one in 40 hospitalizations in 2007 [3]. It has long been apparent that host genetic factors can significantly influence mortality from infectious disease [4], but despite extensive research very few robust associations between genetic polymorphisms and sepsis phenotypes have been reported to date [5]. Possible explanations for these inconsistent findings include inadequate sample sizes (and hence a lack of study power), unidentified population substructure, failure to correct for multiple testing, variations in phenotype definition, heterogeneous patient populations and unrecognized host-pathogen interactions. Nevertheless, we recently published a genome-wide association study (GWAS) in which we identified a common single nucleotide polymorphism (SNP) in the *FER* gene that protects against death from sepsis caused by severe pneumonia [6].

*MBL2* is one of the most extensively studied but still controversial candidate genes reported to be associated with sepsis and infectious disease phenotypes [7, 8]. More than 30 studies have reported the presence or absence of associations between polymorphisms in the *MBL2* gene, circulating MBL levels, and susceptibility and outcomes in sepsis phenotypes, with sample sizes ranging from 6 to 848 patients [8–10].

The *MBL2* gene encodes mannose-binding lectin, which binds carbohydrates on the surface of a wide variety of bacteria, viruses, yeasts, fungi, and protozoa and subsequently triggers host innate immune responses through activation of the complement system, in addition to promoting opsono-phagocytosis by a complement-independent pathway [11]. The function of MBL is known to be influenced by common SNPs found both in the promoter region and in exon 1 of this gene, which affect the level of gene expression and the structure of the MBL protein respectively [12–18].

Three functional SNPs (denoted B-rs1800450, C-rs1800451, and D-rs5030737) in exon 1 of *MBL2* affect the stability, ligand binding capacity, complement activating ability and half-life of the encoded protein [12–15]. Because their effect on serum MBL levels is very similar, wild-type alleles for these SNPs are denoted A, whereas the B, C, and D variants are pooled and given the designation O (Supplementary Figure 1). If an individual is heterozygous (A/O) for any one of these 3 variants, MBL levels are 10%–20% that of wild-type individuals, whereas in variant homozygotes or compound heterozygotes (O/O), serum MBL concentrations are virtually undetectable [16]. *MBL2* promoter polymorphisms have also been associated with MBL levels independent of these exonic SNPs. Allele X of the SNP denoted X/Y at position -221 (rs7096206) has the strongest down-regulating effect [12, 17, 18] and is therefore the most important SNP to analyze, but some studies have also analyzed another promoter SNP, rs11003125 [8, 19].

The aim of this study was to confirm or refute an association between common *MBL2* genetic variants and sepsis susceptibility or outcome. We performed an association study in 1839 patients with sepsis due to community acquired pneumonia (CAP) or peritonitis recruited from intensive care units (ICUs) across Europe as part of the GenOSept and GAinS collaborations and used 477 UK controls recruited within the GRACE consortium. To our knowledge, this is the largest study investigating the influence of *MBL2* polymorphisms on sepsis susceptibility and survival.

## METHODS

### Samples

#### Sepsis Patients (GenOSept and GAinS Consortia)

Patients admitted to critical care units with sepsis caused by CAP or peritonitis were recruited to the ESICM/ECCRN GenOSept (Genetics of Sepsis and Septic Shock in Europe) study between June 2006 and October 2009 from 143 centers in 16 European countries. Recruitment as part of the GAinS study (part of which was included in the GenOSept sample set) began in September 2005 and continued in UK centers, using the same protocol, after the GenOSept study was closed; UK samples from patients recruited to GAinS until October 2010 were also included in this study. Ethics approval was granted either nationally, for individual centers or both. In all cases written, informed consent was obtained from the patient or a legal representative. Diagnosis of sepsis was based on the International Consensus Criteria published in 2003 [20]. Inclusion criteria were adult patients (≥18 years) admitted to an ICU or High Dependency Unit with CAP or peritonitis. The diagnosis of CAP was based on a febrile illness associated with cough, sputum production, breathlessness, leukocytosis, and radiological features of pneumonia acquired within the community or within less than 2 days of hospital admission [21]. Exclusion criteria at recruitment were: pregnancy, an advanced directive to withhold or withdraw life sustaining treatment, admission for palliative care only, and immune-compromise. These collections are also described in more detail elsewhere [6, 22, 23].

Microbiological investigations were performed according to local policies and practices. Investigators recorded microbiological findings for patients diagnosed with CAP, including the organism(s) isolated, the source of the organism and the use of serological methods. The local investigators recorded whether or not initial antibiotic therapy (within the first 24 hours) was considered to be appropriate. Death or survival was recorded at ICU discharge, hospital discharge, and 6 months from ICU admission. The date of death was also recorded.

DNA was extracted by GenOSept partners in London and Munich with a salting out method, in Paris with MagNA Pure Compact DNA Isolation Kits, and in Oxford with Qiagen Midi kits.

#### UK Controls (GRACE Network)

Controls were recruited as part of the GRACE network study (www.grace-lrti.org) [24] and were previously used in the analysis of susceptibility to lower-respiratory tract infections (results submitted for publication). They were individuals attending general practice surgeries for reasons other than infection. All controls included in this analysis were of self-reported Caucasian ancestry from the United Kingdom. DNA was extracted by Maxwell (Promega) at the Wellcome Trust Centre for Human Genetics, University of Oxford.

### Genotyping

The 3 exonic SNPs were successfully genotyped in 1839 sepsis samples by using high-resolution melting analysis (HRMA), with the assays described by Vossen et al [25]. Promoter polymorphism -221 genotypes for the sepsis samples were mainly generated with the Affymetrix 5.0 genotyping chip (N = 1477), as part of the genome-wide association study reported elsewhere [6]. The rest of the sepsis samples (N = 369) were genotyped by HRMA as above. All the healthy controls (N = 477) were genotyped by HRMA. The genotypes of 19 samples were confirmed using direct sequencing. Further details of the genotyping techniques, quality control (QC) procedures, and sequencing can be found in the Supplementary Information.

### Statistical Analysis and Subgroups

#### Genotyping QC

Genotypes for all 4 SNPs obeyed Hardy–Weinberg equilibrium with a *P*-value threshold of .05. The majority of the samples have been genome-wide genotyped; therefore we were able to exclude population outliers (40 individuals) and check for relatedness among individuals (no related individuals were detected). GWAS QC was performed as described elsewhere [6].

#### Possible Confounding Factors

Possible confounding factors were tested for their association with outcome, using analysis of variance (for age) and the χ^2^ test (for other factors) in SPSS v18 (PASW Statistics 18). Age associated significantly with 28-day mortality from sepsis (*P* = 6.25 × 10^−21^), whereas other possible confounding factors such as gender (*P* = .697), CAP/peritonitis diagnosis (*P* = .191), and presence of comorbid respiratory disease (*P* = .317) did not. Therefore, age was included as a covariate in all the analyses.

#### Sepsis Outcome Analyses

All analyses were performed by logistic regression with age as a covariate using SPSS v18 (PASW Statistics 18). We chose our primary end-point for survival analyses as 28 days from ICU admission, because most studies of sepsis patients have used 28-day mortality as the primary end-point to reflect “attributable mortality” [26–28]. We also analyzed longer term survival at 6 months. Although we included age as a covariate in the primary analyses, we also looked for associations in 2 groups of younger patients (less than 70 and less than 60 years of age) in whom it might be expected that MBL deficiency would play a greater role. We performed analyses of associations with outcome in the UK individuals only, to account for population bias. In addition, we analyzed survival in the more homogeneous cohorts of patients with sepsis caused by CAP alone, sepsis caused by peritonitis alone and CAP sepsis caused by *Streptococcus pneumoniae* (the largest subgroup defined by organism).

#### Sepsis Susceptibility Analyses

In the susceptibility analyses, we included only UK patients to compare to the UK controls and examined associations with susceptibility to CAP sepsis and CAP sepsis caused by *S. pneumoniae*.

#### Genotype Combinations Tested for Associations

We performed analyses defining deficient and sufficient genotypes in 3 different ways (by defining O/O, XO/O and XA/O combined genotypes, O/O, XO/O, XA/O and XA/XA combined genotypes [16] or O/O, XO/O, XA/O and YA/O combined genotypes [10] as MBL deficient and all other combined genotypes as MBL sufficient). We also performed analyses for each combined genotype compared to the wild-type YA/YA combined genotype and for the distribution of all 6 combined genotypes (O/O, XA/O, XA/XA, XA/YA, YA/O, and YA/YA). The allelic, genotypic, recessive, dominant, and heterozygous tests were also performed on each SNP individually, and on the AA, AO, and OO combined exon genotypes.

#### Power Calculations

This study had 80% power to detect odds ratios (ORs) of 1.45, 1.57, and 1.52 or more in the analysis of 28 day survival, 6 month survival, and CAP sepsis susceptibility, respectively. Even in our most underpowered subgroup (28 day survival in those with pneumococcal CAP sepsis) we had 80% power to detect ORs of 2.5 or more.

#### Correction for Multiple Testing

Because we have analyzed 4 SNPs in this study, Bonferroni correction gives a *P*-value threshold of .0125 for an association to be declared significant. Given that we have performed analyses using many different models and phenotypes, however, this correction may not be considered to be sufficiently stringent.

## RESULTS

We recruited and analyzed 1839 patients with sepsis (983 with CAP, 856 with peritonitis). The median APACHE score was 17 (range 2–44), and the mortality rate was 19.4%. The majority of patients were recruited in the United Kingdom (Table 1). Demographic and clinical characteristics of the enrolled patients (cases and controls) are shown in Table 1.

**Table 1.** Demographic Data for Patients and Controls

	Sepsis Cases	Control Samples
N	1839	477
N deaths at 28 d	357 (19.4%)	0
Males	1069 (58.1%)	189 (39.6%)
Mean age (standard deviation)	64.2 (15.3)	49.2 (18.7)
N CAP	983 (53.5%)	NA
N peritonitis	856 (46.5%)	NA
APACHE II score; median (range)	17 (2–44)	NA
*S. pneumoniae* identified (CAP samples only)	245 (13.3%)	NA
Respiratory disease	642 (34.9%)	NA
COPD	296 (16.1%)	10 (2.1%)
Asthma	140 (7.6%)	59 (12.4%)
United Kingdom	831	447
Spain	228	NA
Belgium	155	NA
Germany	153	NA
Czech Republic	109	NA
Italy	97	NA
Poland	92	NA
France	59	NA
Ireland	54	NA
Estonia	22	NA
Serbia and Montenegro	21	NA
Greece	5	NA
Croatia	5	NA
Hungary	5	NA
Israel	2	NA
Netherlands	1	NA

Abbreviations: APACHE, Acute Physiology, Age, Chronic Health Evaluation; CAP, community-acquired pneumonia; COPD, chronic obstructive pulmonary disease; NA, not applicable.

The observed allele frequencies in the sepsis cases and UK controls were comparable to those observed in previously published, large population-based European studies (Table 2) [9, 29]. Age was included as a covariate in all analyses. The antibiotics administered within the first 24 hours were considered to be adequate in 90.2% of cases, whereas in 6.5% of patients initial antimicrobial treatment was considered to be inappropriate and for 3.4% data was not available. There was no difference in the proportion of patients receiving adequate antibiotic therapy between survivors and nonsurvivors (*P* = .773) or between individuals who were MBL sufficient or deficient (*P* = .275) (based on the most commonly used definition determined by genotype combinations).

**Table 2.** *MBL2* Allele Frequencies in the Study Reported Here and 2 Large Published European Studies

	Sepsis Cases	UK Controls	Dahl et al [29]	Garcia-Laorden et al [9]		
N samples	**1839**	**477**	**9245**	**848 CAP**	**519 controls**	**1447 healthy controls**
Country of samples	Europe-wide	UK	Denmark	Spain	Spain	Spain
B allele frequency	0.149	0.151	0.145	0.159	0.139	0.142
C allele frequency	0.019	0.024	0.017	0.022	0.031	0.029
D allele frequency	0.070	0.070	0.076	0.047	0.045	0.059
X allele frequency	0.219	0.203	Not genotyped	0.194	0.227	0.206

### Survival Analysis

*MBL2* genotype distributions for the main survival analyses are presented in Table 3. MBL genotypic functional status (where the O/O, XO/O, and XA/O combined genotypes were defined as MBL deficient and all other combined genotypes as MBL sufficient) did not associate with 28-day mortality (*P* = .17), 6-month mortality (*P* = .23) or 28-day pneumococcal CAP mortality (*P* = .64). No significant associations were seen in any of the other subgroups (*P* > .05 for all subgroups shown in Table 3).

**Table 3.** MBL Genotypic Functional Status and the Combined Genotype Distribution (−221 Promoter Single Nucleotide Polymorphism [SNP] and 3 Functional Exonic SNPs) Among Survivors and Deaths in 28-day and 6-month Survival Analyses, and in 28-day Pneumococcal Survival Analysis

MBL Functional Status	Genotypes	CAP and Peritonitis Sepsis 28 d Deaths	CAP and Peritonitis Sepsis 28 d Survivors	CAP Sepsis 28 day Deaths	CAP Sepsis 28 d Survivors	Peritonitis Sepsis 28 d Deaths	Peritonitis Sepsis 28 d Survivors	Pneumococcal Sepsis 28 d Deaths	Pneumococcal Sepsis 28 d Survivors	Sepsis 6 mo Deaths	Sepsis 6 mo Survivors
MBL Deficient	XO/O (%)	1 (0.3)	1 (0.1)	0	1 (0.1)	1 (0.6)	0	0	0	0	1 (0.1)
	O/O (%)	19 (5.3)	81 (5.5)	6 (3.4)	43 (5.3)	13 (7.3)	38 (5.6)	2 (4.4)	11 (5.5)	12 (4.5)	35 (5.0)
	XA/O (%)	30 (8.4)	162 (10.9)	18 (10.1)	84 (10.4)	12 (6.7)	78 (11.5)	5 (11.1)	24 (12.0)	28 (10.6)	73 (10.4)
	**Total (%)**	**50 (14.0)**	**244 (16.5)**	**24 (13.5)**	**128 (15.9)**	**26 (14.5)**	**116 (17.1)**	**7 (15.5)**	**35 (17.5)**	**40 (15.1)**	**109 (15.5)**
MBL Sufficient	XA/XA (%)	17 (4.8)	75 (5.0)	6 (3.4)	40 (5.0)	11 (6.1)	35 (5.2)	1 (2.2)	15 (7.5)	10 (3.8)	34 (4.8)
	XA/YA (%)	74 (20.7)	355 (24.0)	42 (23.6)	198 (24.6)	32 (17.9)	157 (23.2)	12 (26.7)	40 (20.0)	67 (25.3)	166 (23.6)
	YA/O (%)	98 (27.4)	381 (25.7)	48 (27.0)	211 (26.2)	50 (27.9)	170 (25.1)	9 (20.0)	57 (28.5)	68 (25.7)	189 (26.8)
	YA/YA (%)	118 (33.1)	427 (28.8)	58 (32.6)	228 (28.3)	60 (33.5)	199 (29.4)	16 (35.6)	53 (26.5)	80 (30.2)	206 (29.3)
	**Total (%)**	**307 (86.0)**	**1238 (83.5)**	**154 (86.5)**	**677 (84.1)**	**153 (85.5)**	**561 (82.9)**	**38 (84.5)**	**165 (82.5)**	**225 (85)**	**595 (84.5)**
	Overall Total	357	1482	178	805	179	677	45	200	265	704
*P* Value for deficient/sufficient status		.169		.198		.585		.643		.229	

Percentages of genotype distributions in each patient group are shown in brackets. Analyses were also performed for 3 subgroups of CAP and peritonitis patients; those under 70, those under 60, and those from the UK. None of the associations in these subgroups were significant (*P*-values .671, .235 and .870 respectively). The 2 detected XO/O genotypes both included one polymorphism in the −221 promoter, and B and D exonic SNPs, and were confirmed by sequencing (Supplementary Figure 2). Association tests (performed by logistic regression with age as a covariate in SPSS version 18) for deficient/sufficient status were nonsignificant for all subgroups.

Abbreviations: CAP, community-acquired pneumonia; MBL, mannose-binding lectin; SNP, single nucleotide polymorphism.

The specific combinations of MBL genotypes that define functional sufficiency or deficiency remain uncertain. Therefore, we also performed sufficient/deficient analyses by defining genotypes XO/O, O/O, XA/O, and XA/XA [16] or XO/O, O/O, XA/O, and YA/O [10] as MBL deficient. There were no significant associations in the whole cohort or for any of the subgroups shown in Table 3 (*P* > .05).

We also performed analyses of each genotype individually, combined genotypes against each other and the exonic genotypes only combined (A/A, A/O, O/O). Genotype frequencies are listed for survivors and nonsurvivors for the main survival subgroups analyzed in this cohort (Table 4). Across all these survival analyses, the lowest observed nominal *P*-value was .017 (Bonferroni corrected significance threshold *P* = .0125), for the promoter SNP heterozygous test in the peritonitis 28-day survival subgroup.

**Table 4.** Exonic *MBL2* Genotypes for the Main Survival Analysis Subgroups

*MBL2* Genotype	Sepsis 28 d Deaths	Sepsis 28 d Survivors	Sepsis 6 mo Deaths	Sepsis 6 mo Survivors	Pneumococcal CAP Sepsis 28 d Deaths	Pneumococcal CAP Sepsis 28 d Survivors
A/A (%)	**209 (58**.**5)**	**857 (57**.**8)**	**157 (59**.**2)**	**406 (57**.**7)**	**29 (64**.**4)**	**108 (54)**
A/B (%)	81 (22.7)	336 (22.7)	58 (21.9)	164 (23.3)	8 (17.8)	46 (23)
A/C (%)	10 (2.8)	43 (2.9)	6 (2.3)	20 (2.8)	0	8 (4.0)
A/D (%)	37 (10.4)	164 (11.1)	32 (12.1)	78 (11.1)	6 (13.3)	27 (13.5)
Sum A/O (%)	**128 (35**.**9)**	**543 (36**.**7)**	**96 (36**.**2)**	**262 (37**.**2)**	**14 (31**.**1)**	**81 (40**.**5)**
B/B (%)	3 (0.8)	39 (2.6)	5 (1.9)	17 (2.4)	0	9 (4.5)
B/C (%)	2 (0.6)	5 (0.3)	0	0	0	0
B/D (%)	9 (2.5)	30 (2.0)	4 (1.5)	16 (2.3)	2 (4.4)	2 (1.0)
C/C (%)	0	1 (0.1)	0	1 (0.1)	0	0
C/D (%)	3 (0.8)	4 (0.3)	2 (0.8)	2 (0.3)	0	0
D/D (%)	3 (0.8)	3 (0.2)	1 (0.4)	0	0	0
Sum O/O (%)	**20 (5**.**6)**	**82 (5**.**5)**	**12 (4**.**5)**	**36 (5**.**1)**	**2 (4**.**4)**	**11 (5**.**5)**
Total	357	1482	265	704	45	200
*P*-value for AA,AO,OO genotypic test	.937		.697		.488	

Percentages of genotype distributions are shown in brackets. In addition to the genotypic test, we have also performed the allelic, recessive, dominant and heterozygous tests for the AA, AO, OO genotypes, and all individual exonic SNPs (all *P*-values were nonsignificant following correction for multiple testing).

Abbreviations: CAP, community-acquired pneumonia; SNP, single nucleotide polymorphism.

### Susceptibility Analysis

Susceptibility analyses were performed only among the individuals from the United Kingdom. CAP sepsis cases (N = 496) and pneumococcal CAP sepsis cases (N = 95) were compared to controls (N = 477) from the United Kingdom recruited within the GRACE study. *MBL2* genotype distributions for the cases and controls in the susceptibility analyses are presented in Table 5. MBL functional status did not associate with CAP sepsis susceptibility (*P* = .343) or with pneumococcal sepsis susceptibility (*P* = .587). As with the survival analyses, we also analyzed functionally sufficient/deficient genotypes according to their alternative definitions. There were no associations in any of these susceptibility analyses (*P* > .05).

**Table 5.** MBL Genotypic Functional Status and the Combined Genotype Distribution (−221 Promoter Single Nucleotide Polymorphism [SNP] and 3 Functional Exonic SNPs) Among Cases and Controls From the United Kingdom in Community-Acquired Pneumonia (CAP) Sepsis and Pneumococcal CAP Sepsis Susceptibility Analyses

MBL Functional Status	Genotypes	UK Controls	UK CAP Sepsis Cases	UK Pneumococcal CAP Sepsis Cases
MBL Deficient	XO/O (%)	0	1 (0.2)	0
	O/O (%)	19 (4.0)	27 (5.4)	6 (6.3)
	XA/O (%)	57 (11.9)	45 (9.1)	12 (12.6)
	**Total (%)**	**76 (15.9)**	**73 (14.7)**	**18 (18.9)**
MBL Sufficient	XA/XA (%)	15 (3.1)	22 (4.4)	6 (6.3)
	XA/YA (%)	107 (22.4)	128 (25.8)	21 (22.1)
	YA/O (%)	139 (29.1)	128 (25.8)	22 (23.2)
	YA/YA (%)	140 (29.4)	145 (29.2)	28 (29.5)
	**Total (%)**	**401 (84.0)**	**423 (85.2)**	**77 (81.1)**
	Overall Total	477	496	95
*P* Value for deficient/sufficient status compared to controls		NA	.343	.587

Percentages of genotype distributions are shown in brackets. Both detected XO/O genotypes included one polymorphism in the −221 promoter, B and D exonic SNPs, and were confirmed by sequencing. Association tests (performed by logistic regression with age as a covariate in SPSS version 18) for deficient/sufficient genotypes for each set of cases compared to the controls were nonsignificant.

Abbreviations: CAP, community-acquired pneumonia; MBL, mannose-binding lectin; NA, not applicable; SNP, single nucleotide polymorphism.

We also performed analyses of each genotype individually, combined genotypes against each other and only the exonic genotypes combined (A/A, A/O, O/O). Genotype frequencies are listed for cases and controls in Table 6. Across these susceptibility analyses, the lowest observed nominal *P*-value was .029 (Bonferroni corrected significance threshold *P* = .0125), for the B exon SNP recessive test in the UK pneumococcal susceptibility subgroup.

**Table 6.** Exonic *MBL2* Genotypes for the Susceptibility Analysis Subgroups

*MBL2* Genotype	UK Controls	UK CAP Sepsis Cases	UK Pneumococcal CAP Sepsis Cases
A/A (%)	**262 (54.9)**	**295 (59.5)**	**55 (57.9)**
A/B (%)	122 (25.6)	114 (23.0)	23 (24.2)
A/C (%)	20 (4.2)	10 (2.0)	2 (2.1)
A/D (%)	54 (11.3)	49 (9.9)	9 (9.5)
Sum A/O (%)	**196 (41.1)**	**173 (34.9)**	**34 (35.8)**
B/B (%)	6 (1.3)	13 (2.6)	5 (5.3)
B/C (%)	2 (0.4)	0	0
B/D (%)	8 (1.7)	10 (2.0)	1 (1.1)
C/C (%)	0	0	0
C/D (%)	1 (0.2)	4 (0.8)	0
D/D (%)	2 (0.4)	1 (0.2)	0
Sum O/O (%)	**19 (4.0)**	**28 (5.6)**	**6 (6.4)**
Total	477	496	95
*P* Value for AA,AO,OO genotypic test	NA	.170	.530

Percentages of genotype distributions are shown in brackets. In addition to the genotypic test, we have also performed the allelic, recessive, dominant and heterozygous tests for the AA, AO, OO genotypes, and all individual exonic SNPs (All *P*-values were nonsignificant following correction for multiple testing).

Abbreviations: CAP, community-acquired pneumonia; NA, not applicable; SNP, single nucleotide polymorphism.

## DISCUSSION

We report a large, comprehensive analysis of associations between the most extensively studied functional *MBL2* polymorphisms and sepsis survival, as well as susceptibility to CAP and pneumococcal pneumonia. Our large sample collection enabled us to also examine *MBL2* genotype associations with survival in a number of predefined homogeneous subgroups, including younger age groups, UK patients only, patients with CAP, those with pneumococcal pneumonia, and patients with peritonitis. Our findings strongly suggest that there are no significant, clinically meaningful associations between MBL genotypes and either 28-day or 6-month survival from sepsis, or susceptibility to CAP or pneumococcal pneumonia in immune competent adults. The lowest *P*-value we observe across all tests for all subgroups is .017 (for the promoter polymorphism heterozygous test in the peritonitis 28-day survival subgroup). This cannot be considered a significant result after even a nonconservative correction for multiple testing. Moreover it is unlikely that population stratification would have confounded our results as we were able to remove population outliers from the analyses based on multidimensional-scaling using genome-wide genotypes.

The effect of *MBL2* functional genetic variants has been studied extensively in a number of sepsis related phenotypes. Garcia-Laorden et al [9] found, in one of the largest studies (Table 2), that *MBL2* genotypes do not associate with susceptibility to CAP in adults, which is consistent with our findings. They also concluded, as we did, that there was no association between 28-day mortality and *MBL2* deficient genotypes. They did, however, find an association with 90-day survival. Our 6-month survival analysis, however, does not support such an association with longer term survival, even though we have >99% power to detect an association with the OR that they observed (OR = 2.34). Some members of our group [30] have previously reported a study with a phenotype similar to ours but recruited fewer samples (174 cases and 353 controls). These investigators found an association between A/O and O/O exonic polymorphisms and susceptibility to sepsis and also showed an even more significant association between the distribution of combined genotypes and susceptibility to sepsis. We did not replicate these findings in this larger sample set.

Eisen et al [31] performed a meta-analysis of 6 studies of MBL polymorphisms in patients with severe bacterial infection. They found an association between low circulating levels of MBL and mortality and an increased risk of death from *S. pneumoniae* infection (OR = 5.62). We did not find an association between *MBL2* deficient genotypes and death from sepsis due to *S. pneumoniae* infection, despite having >99% power to find an association of this strength. However, Eisen et al also showed that *MBL2* genotype is not a reliable predictor of MBL levels, with the proportion of MBL deficient individuals being underestimated when defined by genotype. It is therefore possible that we did not capture all MBL deficient individuals, and that low MBL levels, rather than genotype, might be associated with worse outcomes in our cohort. Nevertheless, our findings strongly suggest that a clinically relevant relationship between *MBL2* genotype and sepsis mortality in immune competent adults is highly unlikely.

A more recent meta-analysis [8] found no evidence of an association between exonic or promoter *MBL2* variants and susceptibility to sepsis in adults. On the other hand, an association was found between exonic polymorphisms and susceptibility to sepsis in paediatric cases. Variant forms of MBL are very common in certain populations among healthy individuals [13], and it is known that alternative immune mechanisms can replace the function of MBL [32] thereby rendering deficient individuals immune competent. This redundancy could explain the lack of a significant association between *MBL2* functional polymorphisms and sepsis susceptibility and outcome in the present study of immune competent adults. MBL plays a particularly important role in host defense in individuals with a compromised or immature adaptive immune system [7]. Our findings do not exclude the possibility that *MBL2* functional polymorphisms significantly influence sepsis susceptibility and outcome in these patient groups.

One of the limitations of our study is that the causal organism was not identified in all cases. Without this information, we have been unable to perform a complete analysis of bacterial and viral subgroups. However, even with this information, subgroups defined by pathogens other than *S. pneumoniae* are likely to be too small to have sufficient power to detect an association.

We identified 2 individuals with variant alleles X, B, and D, which were confirmed by sequencing (Supplementary Figure 2). Previous reports have suggested that due to linkage disequilibrium between the exon and promoter SNP, only the Y allele in the promoter is present with exonic polymorphisms [9, 17]. Our study shows that other combinations are also possible, although they are very rare.

Although the influence of MBL levels and genotypes on sepsis susceptibility and outcome have been extensively investigated, most studies have been underpowered and therefore it is not surprising that findings have been inconsistent. Moreover, it is well recognized that performing meta-analyses using published data are often limited by publication bias and hence an overestimation of the importance of the SNP or SNPs of interest. In this study, we found no significant associations between *MBL2* genotypes and susceptibility to, or survival from sepsis in a sample set that has adequate statistical power to show associations with effect sizes previously reported in independent studies.

## Supplementary Data

Supplementary materials are available at *Clinical Infectious Diseases* online (http://cid.oxfordjournals.org). Supplementary materials consist of data provided by the author that are published to benefit the reader. The posted materials are not copyedited. The contents of all supplementary data are the sole responsibility of the authors. Questions or messages regarding errors should be addressed to the author.

Supplementary Data
