# Supplementary material for: Variants in the Mannose-binding Lectin Gene MBL2 do not Associate With Sepsis Susceptibility or Survival in a Large European Cohort
Source: Clin Infect Dis. 2015 May 12;61(5):695–703. doi: 10.1093/cid/civ378 (PMC4530723; doi:10.1093/cid/civ378)
Supplement: Supplementary Data [file supp_61_5_695__index.html]

Variants in the mannose-binding lectin gene MBL2 do not associate with sepsis susceptibility or survival in a large European cohort — Variants in the Mannose-binding Lectin Gene MBL2 do not Associate With Sepsis Susceptibility or Survival in a Large European Cohort — Variants in the Mannose-binding Lectin Gene MBL2 do not Associate With Sepsis Susceptibility or Survival in a Large European Cohort — Supplementary Data 

# Variants in the Mannose-binding Lectin Gene *MBL2* do not Associate With Sepsis Susceptibility or Survival in a Large European Cohort

## Supplementary Data

Supplementary Data

- Supplementary Data - Docx file
