# Supplementary material for: Variants in the Mannose-binding Lectin Gene MBL2 do not Associate With Sepsis Susceptibility or Survival in a Large European Cohort
Source: Clin Infect Dis. 2015 May 12;61(5):695–703. doi: 10.1093/cid/civ378 (PMC4530723; doi:10.1093/cid/civ378)
Supplement: Supplementary Data [file supp_civ378_civ378supp.docx]

**Supplementary methods**

**Genotyping**

We genotyped 2172 CAP and FP sepsis samples in this study. Some samples were removed from the analysis: 67 because they failed quality control (QC) during our GWAS, 82 because the exon or promoter SNP genotyping in these samples failed, 173 were not analysed because the patient’s survival status was unknown, and 11 samples were withdrawn from the study. There were 1839 samples remaining for analysis. Further details about GWAS QC are provided in our GWAS publication.[^1^](#_ENREF_1)

The three exonic SNPs were genotyped in 1846 CAP and FP sepsis samples using high resolution melting analysis (HRMA), as these SNPs were not genotyped as part of the GWAS chip. Seven samples failed genotyping. Genotypes of eleven samples were confirmed by sequencing. All of these samples were also genotyped for the -221 promoter SNP; 369 by HRMA and 1477 with the genome-wide Affymetrix 5.0 SNP array. Every UK sample in the GRACE study was successfully genotyped for all four MBL SNPs described here, by HRMA, and sequencing to confirm exon genotypes in eight samples.

Affymetrix 5.0 genotyping chip

The majority (1477) of samples in this study were genotyped for the -221 promoter SNP (rs7096206) as part of a genome-wide association study (GWAS) of sepsis outcome.[^1^](#_ENREF_1) Genotyping was carried out in Munich, Germany, with the Affymetrix GeneChip® Genome-Wide SNP 5.0 array. Genotypes were called with the BRLMM-P calling algorithm (Genotyping console v.3, Affymetrix inc.). 187 individuals were excluded following QC procedures; a sample call rate of 98% and SNP call rate of 98% were applied. Population outliers (based on multidimensional scaling), heterozygosity outliers (>3x standard deviation from the mean), related individuals, and individuals with incorrect gender were removed.

High Resolution Melting Analysis (HRMA)

Two HRMA assays were used to genotype four SNPs in the *MBL2* gene, as described by Vossen *et al.*[^2^](#_ENREF_2). Samples of known genotype (confirmed by sequencing) were included on every plate for exon genotyping, as controls. Genotypes were assigned by 96-well plate. All sepsis samples (survivors and non-survivors) were mixed on the same plates when genotypes were assigned. The investigator calling genotypes was blinded to phenotype. HRMA genotyping was performed at the Wellcome Trust Centre for Human Genetics, University of Oxford.

Direct sequencing of *MBL2* exon and promoter polymorphisms

Primers for exon polymorphism sequencing were GCACCCAGATTGTAGGACAGAG (F) and CAGGCAGTTTCCTCTGGAAGG (R) [^3^](#_ENREF_3). PCR conditions were: 94°C for 14 minutes, 38 cycles of 94°C for 30 seconds, 58°C for 30 seconds and 72°C for 30 seconds, then 72°C for 5 minutes.

PCR was performed in a 10µl reaction with final concentrations of 1.2x PCR buffer (Qiagen), 1.2mM MgCl_2_ (Qiagen), 240µM each dNTP (BioLine), 240µM of each primer (Metabion), 0.3 units HotStar Taq DNA Polymerase (Qiagen) and 20ng DNA template. 6µl PCR product was run on a 2% (w/v) Ethidium Bromide agarose gel for 1 hour to check that PCR was successful.

ExoSAP was used to clean PCR products, which were then subject to sequencing reactions. 4µl PCR product was treated with 1 unit of Shrimp Alkaline phosphatase (Promega) and 2 units of Exonuclease I (New England Biolabs) in a final volume of 8µl with 0.5x Shrimp alkaline phosphatase buffer (Promega).

2µl purified PCR product was used in a 10µl reaction containing 0.25µl Big Dye Terminator Sequencing Mix v3.1 (ABI), 0.94x Big Dye Dilution Buffer, 0.5µM primer (forward or reverse depending on which direction the reaction is for). Primers were the same as those used for PCR. Sequencing conditions were as follows: 25 cycles of 96°C for 10 seconds, 50°C for 5 seconds and 60°C for 4 minutes.

Ethanol precipitation was used to clean products, which were then run on an ABI 3730xl capillary sequencer, in the Zoology department at the University of Oxford. Sequence analysis was performed using Sequencher 4.2 software. Traces were checked for any unreliable calls, and forward and reverse strands were aligned. The genotype of the SNPs was manually checked on chromatograms.

**Supplementary Figures**


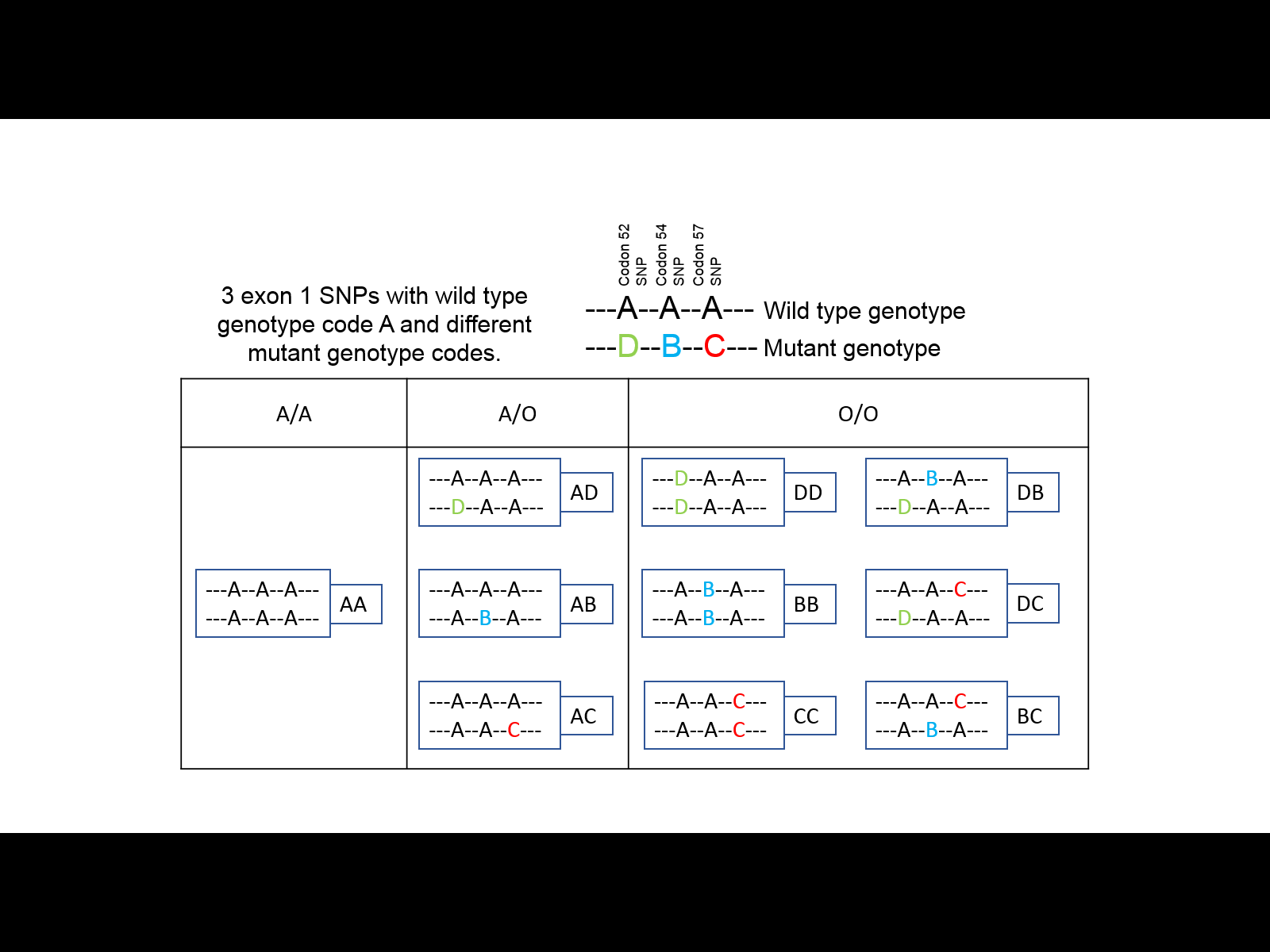


**Supplementary Figure 1**

*MBL2* exon 1 genotypes and their designation when combined for analysis. The three functional exon 1 SNPs in the MBL2 gene are rs5030737 (denoted D, codon 52, C/T SNP), rs1800450 (denoted B, codon 54, G/A SNP) and rs1800451 (denoted C, codon 57, G/A SNP). Heterozygotes for one SNP (AD, AB or AC) are grouped and designated A/O. Homozygotes for one SNP (DD, BB or CC) and heterozygotes for two of the SNPs (DB, DC or BC) are grouped and designated O/O.

(A)
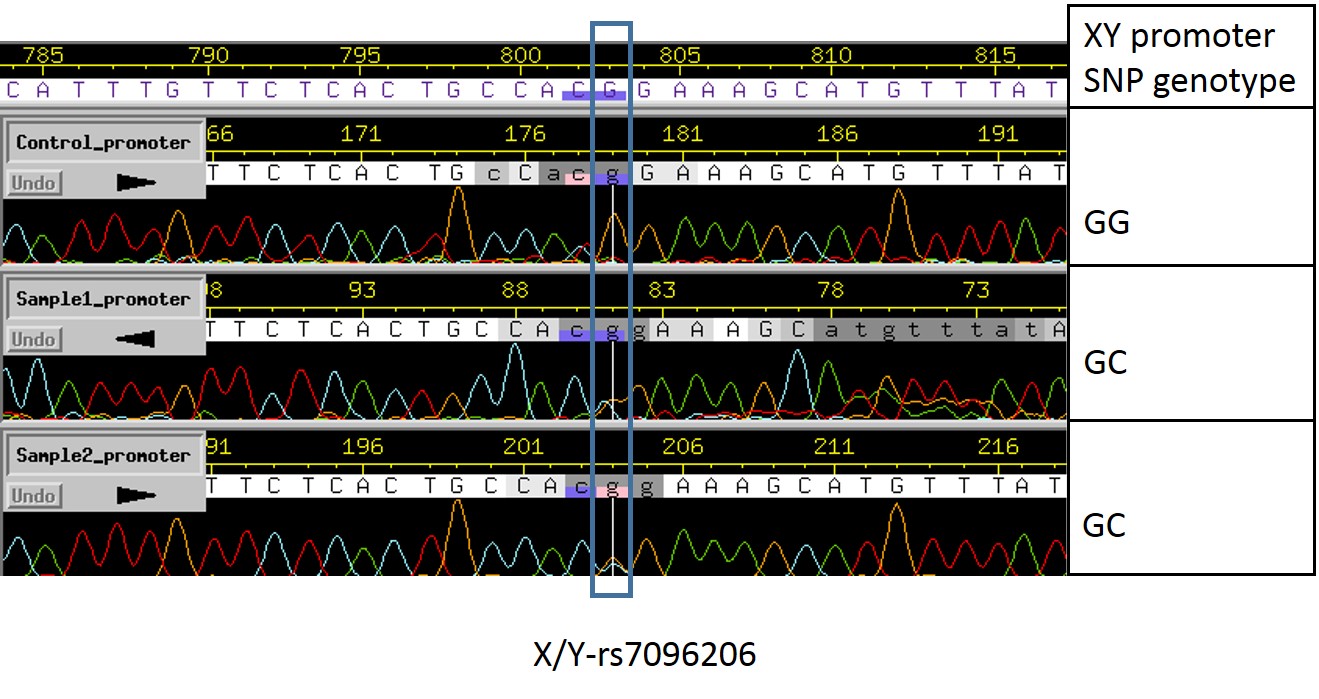


(B)


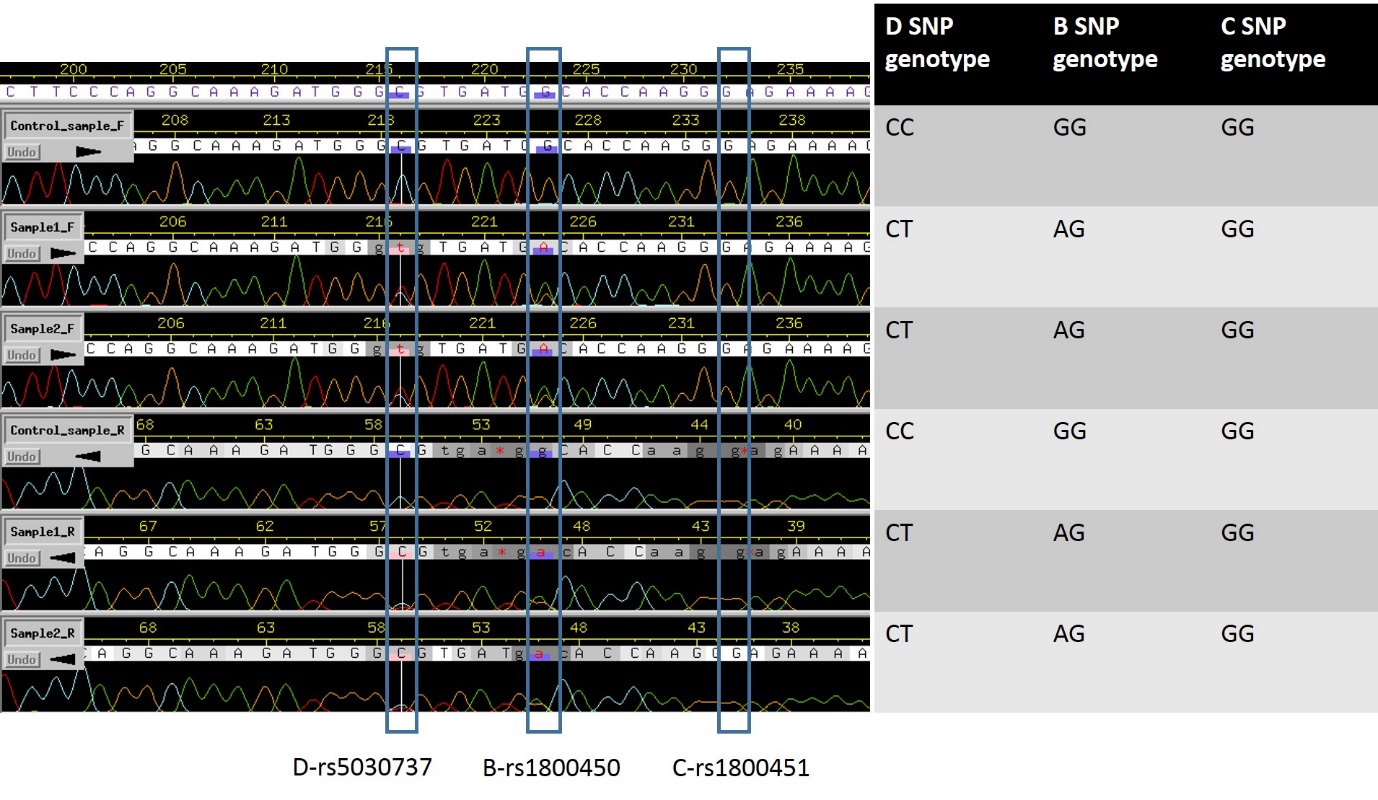


**Supplementary Figure 2**

Chromatograms for one control sample (where all 4 SNPs of interest have wild type calls) and the two samples which have XBD genotypes. (A) Promoter region Sanger sequencing trace results. The XY promoter SNP (rs7096206) is highlighted by a blue box. Samples 1 and 2 show heterozygote calls at this position, whereas the control sample shows a GG wild type homozygote. A previously undescribed SNP is observed at the position before rs7096206 in the control sample. (B) Exon region Sanger sequencing trace results. The 3 exonic SNPs are highlighted with blue boxes and labelled with their letter code and rsID. Genotypes for each SNP and for each sample are shown in the table on the right. Traces are shown for sequencing in both directions in this region. Samples 1 and 2 show heterozygote calls for the D and B SNPs, while all other SNPs are wild type homozygotes in this region.

1. Rautanen A, Mills TC, Gordon AC, et al. Genome-wide association study of survival from sepsis due to pneumonia: an observational cohort study. *The Lancet Respiratory medicine* 2015; **3**(1): 53-60.

2. Vossen RHAM, Aten E, Roos A, den Dunnen JT. High-Resolution Melting Analysis (HRMA) - More than just sequence variant screening. *Human Mutation* 2009; **30**(6): 860-6.

3. Chapman S, Vannberg F, Khor C, et al. Mannose-binding lectin genotypes: lack of association with susceptibility to thoracic empyema. *BMC Medical Genetics* 2010; **11**(1): 5.
